# Reconstruction of Imagined Melody with Relative Pitch Decoding in Electrocorticography

**DOI:** 10.1523/ENEURO.0289-25.2026

**Published:** 2026-08-06

**Authors:** Jii Kwon, Youmin Shin, June Sic Kim, Eunju Jeong, Sung-Phil Kim, Eun Jung Lee, Chun Kee Chung

**Affiliations:** ^1^Department of Brain & Cognitive Sciences, Seoul National University, Seoul 08826, Republic of Korea; ^2^Seoul AI School, Seoul School of Integrated Sciences and Technologies (aSSIST) University, Seoul 03767, Republic of Korea; ^3^Research Institute of Basic Sciences, Seoul National University, Seoul 08826, Republic of Korea; ^4^Department of Music Therapy, Graduate School, Ewha Womans University, Seoul 03760, Republic of Korea; ^5^Department of Biomedical Engineering, Ulsan National Institute of Science and Technology, Ulsan 44919, Republic of Korea; ^6^Department of Neurosurgery, Seoul National University Hospital, Seoul National University College of Medicine, Seoul 03080, Republic of Korea; ^7^Neuroscience Research Institute, Seoul National University College of Medicine, Seoul 03080, Republic of Korea

**Keywords:** electrocorticography, music imagery, neural decoding, relative pitch, superior temporary gyrus

## Abstract

Music imagery involves internally generated auditory experiences, yet decoding imagined melodic content from neural activity remains challenging. Here, we examined whether imagined melodies can be decoded from human electrocorticography (ECoG) recordings using a relative pitch framework. Ten epilepsy patients performed a music imagery task involving familiar melodies presented in multiple tonalities (3 males and 7 females; mean age, 27.3 ± 4.69 years). Neural features from imagery-responsive cortical sites were used to train intrasubject models to decode relative pitch classes at the single-note level. Although single-note decoding accuracy was modest, it reliably exceeded chance. Sequential integration of decoded pitch classes enabled reconstruction of melodic contours that preserved the relative structure of imagined melodies. These results provide a proof-of-concept that imagined melodic structure can be accessed from intracranial neural recordings when framed in terms of relative pitch, underscoring the importance of sequence-level approaches for decoding internally generated auditory content.

## Significance Statement

Imagining music is a rich internal experience, yet decoding the content of imagined melodies from brain activity remains a major challenge. Most previous studies have focused on detecting music imagery or decoding coarse musical features, rather than reconstructing melodic structure itself. In this study, we demonstrate that imagined melodies can be decoded from human intracranial brain recordings by representing melody in terms of relative pitch, a fundamental principle of musical perception. Although decoding individual imagined notes is difficult, combining note-level predictions over time allows recovery of melodic contours that preserve the structure of imagined songs. These results show that structured internal musical representations can be accessed from neural signals and suggest a principled pathway toward decoding complex imagined auditory content.

## Introduction

Melody is a fundamental component of music and can be defined as an ordered sequence of pitches characterized by their relative intervallic relationships (Gómez et al., 2003). It provides a distinctive identity that allows listeners to recognize and remember musical pieces across transpositions and changes in timbre. However, despite growing interest in music imagery, attempts to decode imagined music by explicitly predicting melodic structure remain scarce.

Neurophysiological studies of music listening indicate that melodic information is supported by a distributed auditory network, prominently involving high gamma activity (HGA) in the superior temporal gyrus (STG; Herff et al., 2020; Sankaran et al., 2024). These regions are implicated in processing pitch height, interval structure, and melodic contour, thereby supporting the extraction of relational pitch information that defines melody (Patterson et al., 2002; Abrams et al., 2025). Intracranial recordings further suggest that higher-frequency activity in auditory cortex tracks task-relevant auditory representations with high temporal precision, providing a useful substrate for decoding approaches (Crone et al., 2001).

Music imagery—the internal experience of hearing music in the absence of external sound—engages neural systems that substantially overlap with those involved in music perception. Neuroimaging studies have reported imagery-related activation across auditory (Halpern, 1989; Zatorre et al., 1996; Halpern et al., 2004), frontal (Zatorre et al., 1996; Halpern and Zatorre, 1999; Herholz et al., 2012), parietal (Leaver et al., 2009; Herholz et al., 2012; Zhang et al., 2017), and sensorimotor regions (Hubbard, 2019; Regev et al., 2021), consistent with the constructive nature of internally generated auditory representations. Engagement of primary auditory cortex appears less consistent across imagery paradigms, whereas higher-order auditory and associative regions show more reliable involvement (Yoo et al., 2001; Halpern et al., 2004; Zhang et al., 2017). These observations motivate intracranial decoding approaches that target distributed cortical activity during imagery.

Recent advances in neural decoding have begun to explore whether imagined musical content can be inferred from brain activity. Using electroencephalography (EEG), magnetoencephalography (MEG), and electrocorticography (ECoG) recordings, prior work has demonstrated the decoding of rhythmic structure, temporal expectations, and coarse spectral features during music imagery (Martin et al., 2018; Di Liberto et al., 2021; Marion et al., 2021; Quiroga-Martinez et al., 2024). Notably, Chung et al. further demonstrated note-level decoding of imagined pitches from EEG using a visually presented piano-keyboard paradigm within a single tonality (Chung et al., 2023). However, reconstructing imagined melodies as a note sequence that remains interpretable—particularly beyond absolute pitch within a fixed tonal context—remains challenging.

A key insight from music cognition is that melodies are primarily represented in terms of relative pitch rather than absolute pitch (McDermott et al., 2008; Leipold et al., 2019). Relative pitch refers to the perception of pitch relationships between successive tones, and behavioral evidence shows that infants and adults rely on relative cues such as melodic contour to recognize melodies across transpositions (Plantinga and Trainor, 2005). This suggests that decoding relative pitch representations may offer a cognitively grounded route to reconstructing imagined melodic structure.

In the present study, we investigate whether imagined melodies can be decoded from human ECoG recordings within a relative pitch framework. To test feasibility under controlled conditions, we focus on well-learned familiar melodies presented in multiple tonalities, enabling relative pitch classes to be examined across transpositions. We identify imagery-responsive cortical sites based on increased HGA and extract neural features to decode relative pitch classes (Do–La) at the single-note level. We then assess whether sequential integration of decoded pitch classes supports reconstruction of melodic contours. Finally, we perform an exploratory cross-subject analysis to identify neural feature patterns that are consistently informative across individuals despite heterogeneous electrode coverage.

## Materials and Methods

### Subjects

We recruited a total of 17 patients with medically intractable epilepsy who had subdural ECoG electrodes implanted for clinical purposes. During the experiment, three subjects were excluded due to difficulties in understanding the experimental paradigm, and four subjects were excluded because they were unable to accurately sing the trained melodies, which was a predefined requirement for task compliance. As a result, data from 10 subjects were included in the final analysis (3 males and 7 females; mean age, 27.3 ± 4.69 years).

The present study analyzed only ECoG signals recorded from subdural electrodes. Both conventional and high-density subdural electrode grids were used (Ad-Tech Medical Instrument; PMT), with electrode diameters of 3 and 2 mm and center-to-center spacing of 10 and 5 mm, respectively. Electrode implantation was determined solely by clinical considerations.

All subjects were non-musicians without formal musical training and reported no history of auditory impairments. The study protocol was approved by the Institutional Review Board of Seoul National University Hospital (C-2002-014-1098), and all subjects provided written informed consent prior to participation. All experimental procedures were conducted in accordance with relevant guidelines and regulations, including the Declaration of Helsinki (see Table 1 for additional demographic and clinical information).

**Table 1.** Subject demographics

Subject ID	Age (year)	Gender	Diagnosis	Electrode laterality	The number of electrodes	Electrode type
Sub1	22	F	TLE	R	56	Conventional and high density
Sub2	29	F	TLE	L	12	Conventional
Sub3	24	M	PLE/OLE	L	62	Conventional and high density
Sub4	28	M	TLE	R	54	Conventional and high density
Sub5	27	F	TLE	L	18	Conventional
Sub6	37	F	TLE	L	42	Conventional
Sub7	31	F	TLE	L	26	Conventional
Sub8	29	F	TLE	L	68	Conventional/high density
Sub9	25	F	TLE	R	56	Conventional/high density
Sub10	21	M	FLE	R	28	Conventional/high density

F, female; M, male; R, right; L, left; TLE, temporal lobe epilepsy; PLE, parietal lobe epilepsy; OLE, occipital lobe epilepsy; FLE, Frontal lobe epilepsy.

### Electrode localization and anatomical labeling

**Figure 1.**
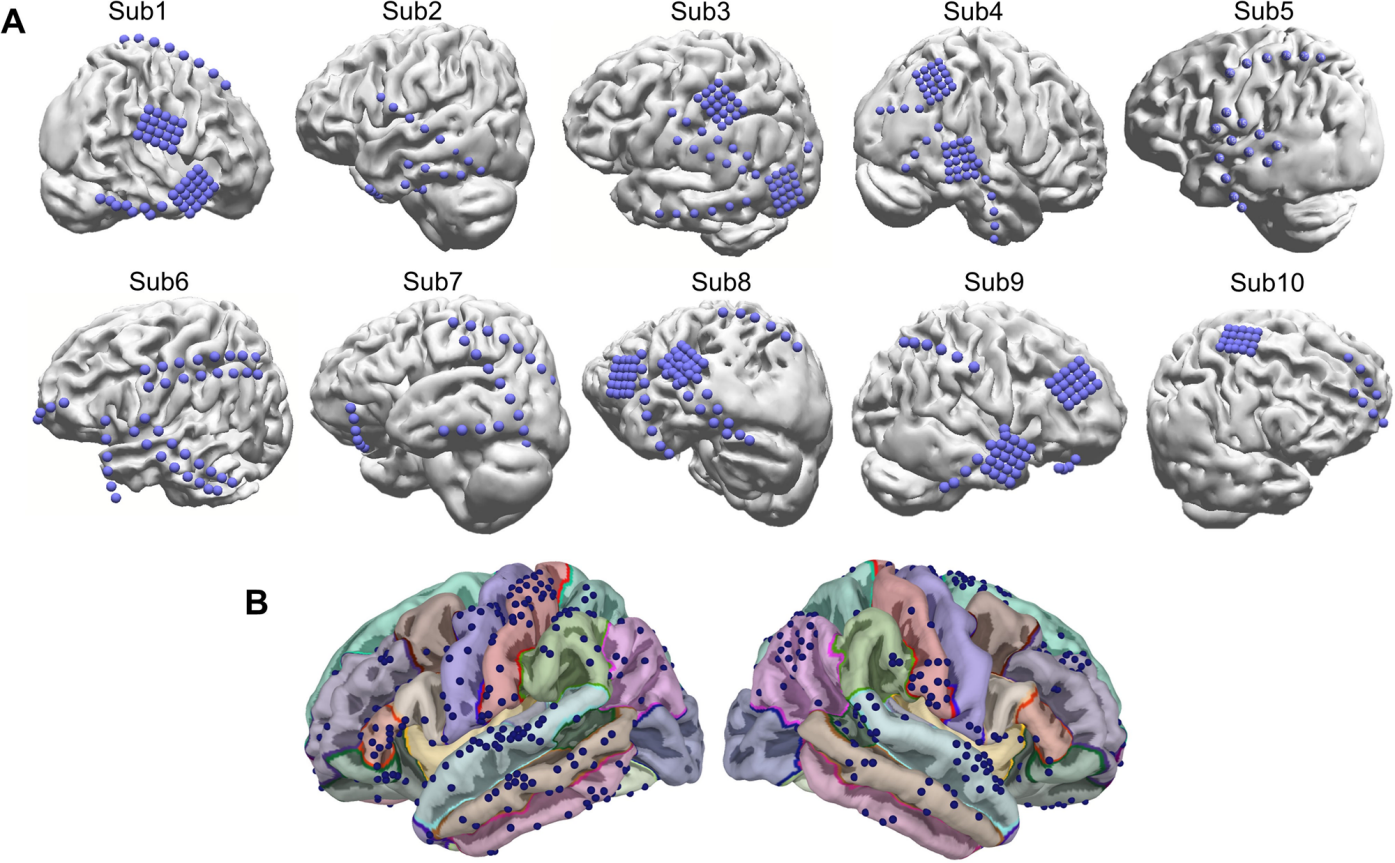
Electrode locations. ***A***, Locations of implanted ECoG electrodes for all subjects shown on individual cortical surfaces. Each dot represents a subdural electrode contact. ***B***, Group-level distribution of electrode locations projected onto an MNI-standardized cortical surface. Dots indicate the locations of implanted electrodes pooled across all subjects. The cortical surface of the template brain is color-coded according to functional regions defined by the Desikan–Killiany cortical parcellation (Fischl et al., 2004), as implemented in the MNE-Python library (Gramfort et al., 2014).

ECoG electrodes were localized individually for each subject by coregistering preoperative magnetic resonance (MR) images with postoperative computed tomography (CT) images using CURRY 9.0 (Compumedics Neuroscan; Fig. 1*A*). MR images were acquired with a Magnetom Trio Tim 3.0 T scanner, and CT images were obtained using a Somatom Sensation 64 eco system (both Siemens). This MR–CT coregistration procedure followed standard clinical and research practices for electrode localization.

Electrode coordinates were initially defined in the Talairach coordinate system and subsequently converted to the Montreal Neurological Institute (MNI) coordinate system using the MATLAB script tal2mni.m from the GingerALE software package (v. 3.0; Lancaster et al., 2007; Laird et al., 2010). Each electrode was then mapped to the nearest cortical surface vertex based on Euclidean distance (Fig. 1*B*), enabling spatial alignment of electrodes across subjects in a common reference space (Kwon et al., 2025).

Anatomical labeling was performed in MNI space using the MNE-Python library (version 1.6.0), which assigned electrodes to Brodmann areas based on their proximity to atlas-defined cortical regions (Gramfort et al., 2013). This atlas-based labeling was used to facilitate group-level anatomical summaries and comparisons across subjects, rather than to assert precise localization at the level of individual cortical areas.

### Experimental design and task

**Figure 2.**
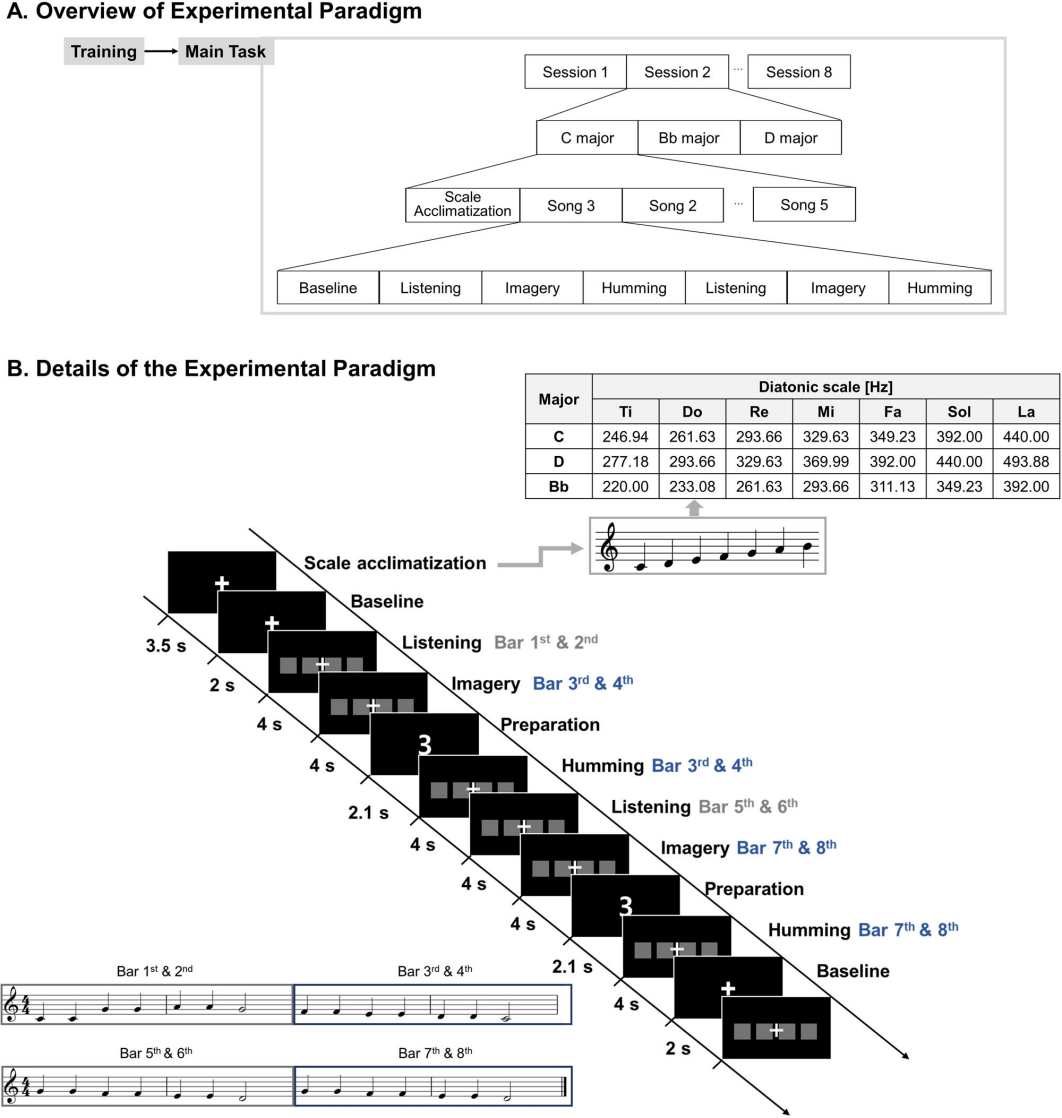
Experimental paradigm. ***A***, Overview of the experimental paradigm. After a training phase, subjects performed the main task, which consisted of eight consecutive sessions. Each session included music listening, music imagery, and humming phases performed with five trained songs presented in three different tonalities. ***B***, Detailed structure of the experimental paradigm. Before each change in tonality, a scale acclimatization phase was presented to familiarize subjects with the diatonic scale of the upcoming key. The table in the top right shows the scale stimuli and their corresponding absolute pitch values for the diatonic scale in C, D, and B♭ major. At the beginning of each song, a baseline fixation period preceded the onset of the first bar. This was followed by alternating phases of music listening and music imagery: subjects listened to the first and second bars (gray panels in the example musical scores at the bottom left) and subsequently imagined the third and fourth bars (blue panels). After a brief preparation phase, subjects hummed the previously imagined bars. This cycle of listening, imagery, preparation, and humming was repeated to cover the fifth through eighth bars of each song. All musical stimuli and visual timing cues were presented at a tempo of 120 bpm. See Extended Data Figure 2-1 for an example of the audio stimulus waveform.

All experiments were conducted within a single recording day for each subject. Each subject completed eight sessions (Fig. 2*A*), which were performed consecutively with short breaks of several minutes between sessions to minimize fatigue. No session involved a separate recording day.

10.1523/ENEURO.0289-25.2026.f2-1 Figure 2-1 **Example of audio stimulus waveform.** The sound stimulus has a duration of 400 ms, including 45 ms of fade-in and fade-out to ensure smooth perception of the pure tone. Download Figure 2-1, TIF file.

Each session consisted of three sequential phases: scale acclimatization, baseline, and main task. During the scale acclimatization phase, subjects were presented with the diatonic scale corresponding to the upcoming tonality, spanning from the leading tone of the lower octave (Ti) to the submediant (La) of the target octave, to facilitate adaptation to the key (Fig. 2*B*). This was followed by a baseline phase, during which subjects fixated on a cross displayed on the screen.

The main task involved five familiar children's songs (Table 2), each presented in three different tonalities (C major, D major, and B♭ major). Familiar melodies were selected to promote stable and accurate music imagery across repeated trials. Within each session, the order of tonalities was randomized, and within each tonality, the order of the five songs was also randomized to minimize order effects and anticipatory strategies.

**Table 2.** Information of music pieces

Index	Song name
S1	*Yankee Doodle*
S2	*Twinkle, Twinkle, Little Star*
S3	*Dragonfly*
S4	*The Ten Little Indians*
S5	*A Harmonica Made of Corn*

Each song consisted of eight bars and followed a structured sequence of music listening, music imagery, and humming. This task shares key structural features with paradigms commonly used in imagined speech studies (Dash et al., 2020). Specifically, subjects first listened to two bars of a song, then imagined the subsequent two bars in silence, and finally hummed the imagined melody after a short preparation period. This sequence was repeated to cover all eight bars of the song.

Music imagery was strictly confined to the imagery phase. Subjects were explicitly instructed to internally imagine hearing the melody while refraining from any overt movement, including vocalization, rhythmic motor actions, or body movement. Visual cues were presented at a tempo of 120 bpm (beats per minute) to temporally align imagery with the expected note onsets. Each imagined note corresponded to a 0.5 s interval, resulting in an imagery window of 4 s per trial (eight notes per trial).

Across a single session, imagery data were collected from five songs presented in three tonalities, yielding multiple imagery trials per scale degree. Throughout the experiment, subjects were continuously monitored by an experimenter to ensure compliance with task instructions and the absence of overt movement during the imagery phase. The humming phase was temporally separated from imagery and occurred only after the imagery interval was completed.

### Sound stimulus

Sound stimuli consisted of pure tones corresponding to the notes of each song, with a duration of 400 ms and an interstimulus interval of 100 ms (Extended Data Fig. 2-1). Pure tones were used to minimize potential confounds arising from timbral variation and to isolate pitch-related information. The stimuli were generated using Audacity (open-source audio editor; Muse Group, International). Each tone included 45 ms linear fade-in and fade-out ramps to ensure smooth perception and to avoid spectral artifacts at onset and offset. The sound stimuli were presented through a speaker (Creative Pebble V2, Creative) placed beneath the monitor ∼50 cm in front of the subject. Sound intensity was adjusted to a comfortable listening level for each subject (∼70 dB SPL). Stimulus presentation was controlled using STIM2 Gentask (Compumedics Neuroscan).

### Neural data acquisition

ECoG signals were recorded using a 128-channel SynAmps2 system (Compumedics Neuroscan) in Subjects 1–5 and a 128-channel Neuvo system (Compumedics Neuroscan) in Subjects 6–10.

Signals were sampled at 2,000 Hz and digitally filtered with a low-pass cutoff frequency of 800 Hz during acquisition. Ground and reference electrodes were positioned adjacent to each other, either at the forehead or cheekbone, depending on individual clinical configurations. Identical preprocessing procedures were applied to data from all subjects irrespective of the recording system.

### ECoG data preprocessing

We excluded the electrodes showing poor signal qualities with high impedance, above 30 kΩ during recordings. The raw data were filtered using a high-pass filter with a 0.1 Hz cutoff frequency for detrending. The filtered data were rereferenced using the common average reference (CAR) to remove global background activity. The rereferenced data were notch-filtered at 60 Hz and its harmonic frequencies.

### Identification of active electrodes

To identify cortical regions involved in music imagery, we compared neural activity during the imagery phase to a baseline fixation period. For each electrode, HGA (61–150 Hz) was averaged across the imagery window (4 s per trial) and compared with the baseline period using a Wilcoxon signed-rank test.

To extract HGA, the complex Morlet wavelet transform was performed with center frequencies spanning 1–200 Hz in 1 Hz increments (200 frequency bins). The wavelet bandwidth was controlled by the Morlet parameter *B* = 2.48 (with center frequency parameter *C* = 1), resulting in frequency-dependent temporal and spectral resolution across the analyzed range. HGA was quantified by averaging normalized power within the 61–150 Hz range during predefined imagery intervals.

Electrodes were classified as active if the mean HGA during music imagery was significantly greater than during baseline fixation (*p* < 0.05). Only electrodes meeting this criterion were included in subsequent feature extraction and decoding analyses. This approach ensured that decoding models were restricted to cortical sites exhibiting imagery-related task engagement.

### Trial structure and data partitioning

Each imagined melody was composed using a diatonic scale, in which each scale degree (Do–Ti) was instantiated by three different absolute pitch values across the three tonalities (Fig. 2*B*). This design ensured that relative pitch classes were represented independently of absolute pitch height, allowing relative pitch representations to be examined across transpositions.

Within a single session, imagery data were collected from five songs presented in three tonalities, yielding multiple note segments per scale degree. The exact number of note segments per scale degree is summarized in Table 3 (on average, 164.5 note segments per class in the training set and 23.5 in the test set). Because the Ti class contained substantially fewer samples than the other scale degrees across sessions, it was excluded from subsequent classification analyses.

**Table 3.** Class distribution in training and test sets

Index	Trials per session	Training set (original)	Training set (after balancing)	Balance strategy	Test set
Do	21	147	157	Oversampling	21
Re	42	294	157	Undersampling	47
Mi	27	189	157	Undersampling	27
Fa	15	105	157	Oversampling	15
Sol	18	126	157	Oversampling	18
La	18	126	157	Oversampling	18
Ti	6	–	–	–	–

The table summarizes the number of trials for each relative pitch class in the training and test sets. Class imbalance in the training set was addressed using oversampling or undersampling, while no data augmentation was applied to the test set. The Ti class was excluded from decoding analyses due to insufficient sample size.

For decoding analyses, data from seven of the eight sessions were used for training and validation, while data from the final (eighth) session were reserved as an independent test set. This session-wise split was adopted to provide a temporally independent evaluation of decoding performance and to minimize potential data leakage between training and test sets. In addition, the final session was performed after subjects had fully adapted to the task structure and stimulus set, making it suitable for assessing melody reconstruction under stable imagery conditions. Model performance was evaluated using sevenfold cross-validation on the training data, resulting in ∼141 training and 23 validation note segments per scale degree per fold. Data augmentation and trial balancing were applied only to the training folds.

### Feature extraction

Neural features were extracted from electrodes that exhibited significant task-related activation during music imagery (see above, Identification of active electrodes). For each selected electrode, the preprocessed ECoG signals were bandpass filtered into six canonical frequency bands—delta (1–4 Hz), theta (4–8 Hz), alpha (8–12 Hz), beta (12–30 Hz), low gamma (LG; 30–59 Hz), and high gamma (HG; 61–150 Hz)—using a finite impulse response bandpass filter implemented with the eegfilt function from the EEGLAB toolbox (Delorme and Makeig, 2004). These frequency bands were selected based on prior electrophysiological studies of cortical oscillations and HGA (Buzsaki and Draguhn, 2004; Crone et al., 2006).

For each frequency band, the analytic signal was computed using the Hilbert transform, and the amplitude envelope was extracted. The envelope signal was then segmented into epochs corresponding to individual imagined notes (0.5 s per note). Within each epoch, envelope values were averaged to yield a single feature value per electrode and frequency band.

### Classification and decoding models

The goal of the decoding analysis was to classify relative pitch classes during music imagery at the level of individual notes. Specifically, each imagery epoch corresponding to a single note (0.5 s) was assigned to one of six relative pitch classes (Do, Re, Mi, Fa, Sol, La). The Ti class was excluded due to an insufficient number of samples, as described above.

Because the number of note segments differed across relative pitch classes, class imbalance was addressed during model training. To mitigate this issue, a combination of oversampling and undersampling was applied exclusively to the training data. Specifically, the Synthetic Minority Over-Sampling Technique (SMOTE) was used to generate synthetic samples for underrepresented relative pitch classes by interpolating between feature vectors of neighboring samples in the feature space (Lee et al., 2020). For classes with a disproportionately large number of samples, random undersampling was applied to reduce their contribution during training. No data augmentation was applied to the independent test set (Table 3).

Decoding analyses were performed using intrasubject models, such that a separate classifier was trained and evaluated for each subject. Input features consisted of band-limited neural activity extracted from electrodes that exhibited significant HGA during music imagery (see above, Identification of active electrodes). For each imagery epoch, features from all selected electrodes and frequency bands were concatenated to form a feature vector representing that note.

We evaluated multiple commonly used classification algorithms, including random forest (RF), support vector machine (SVM), linear discriminant analysis (LDA), quadratic discriminant analysis (QDA), and nearest centroid classifier (NCC). Model selection was performed independently for each subject using a sevenfold cross-validation procedure on the training data. For each subject, the combination of classifier and feature subset yielding the highest mean validation accuracy was selected as the optimal intrasubject decoding model.

After model selection, decoding performance was evaluated on an independent held-out test set comprising data from the final session, which was not used during cross-validation or model optimization. Decoding accuracy was defined as the proportion of correctly classified note epochs, and chance level (CL) was set to 16.7%, corresponding to six classes.

### Increment value and feature contribution analysis

To quantify the contribution of individual neural features to decoding performance, we performed a feature contribution analysis based on an increment value metric. While standard feature importance measures derived from classifier such as RF provide relative rankings, they do not directly indicate how much each feature improves decoding accuracy when incorporated into the model.

The increment value was therefore defined as the increase in decoding accuracy associated with the sequential inclusion of a given feature during a sequential forward selection (SFS) procedure (Mayer et al., 2018; Shin et al., 2023). SFS was implemented using a forward selection approach. Starting from an empty feature set, features were added one by one according to their importance ranking of RF, and decoding performance was evaluated after each addition using cross-validation. This procedure was repeated independently for each classifier to determine the optimal number of features and model type.

For the first selected feature, the increment value was defined as the difference between the decoding accuracy achieved using the feature alone and the CL (Pencina et al., 2012). For subsequent features, the increment value was calculated as the increase in accuracy relative to the model containing the previously selected features, normalized by the number of features included at that step. This procedure yielded a quantitative measure of how much each feature contributed to improving decoding performance.

Importantly, the increment value reflects feature contribution to decoding performance, rather than a causal or mechanistic role in neural encoding. Accordingly, increment values were used to compare the relative utility of features for classification and to identify features that consistently enhanced decoding accuracy across subjects, while avoiding overinterpretation in terms of neural representation.

### Cross-subject decoding analysis

In addition to intrasubject decoding, we conducted an exploratory cross-subject decoding analysis to examine whether common neural features supporting relative pitch imagery could be identified across individuals.

To address variability in electrode coverage across subjects, channel-level features were aggregated within each subject into region-band features. Anatomical labels were assigned based on the electrode localization procedure described above, Electrode localization and anatomical labeling. For each subject, all electrodes belonging to the same anatomical region and frequency band were averaged at the trial level, yielding a single feature for each region-band combination. Thus, one representative region-band feature was generated by averaging all available electrodes within that region-band, rather than by selecting a single electrode. If a subject did not have electrode coverage for a given region-band feature, that feature was treated as missing.

The region-band features used for this analysis were selected based on the top-ranked features from the increment value analysis. Specifically, the top 10 region-band combinations were entered cumulatively according to their ranking: the models was first trained using the highest-ranked feature, then using the top two features, and so on up to the top 10 features. This cumulative procedure was used to evaluate how decoding performance changed as additional shared region-band features were included.

Decoding performance was evaluated using leave-one-subject-out cross-validation. For each fold, the model was trained on trials from all subjects except one and tested on the held-out subject. Missing region-band features were imputed using the mean estimated from the training subjects only. Standard scaling was also fitted only on the training data and then applied to the held-out subject. Therefore, the held-out subject was not used for feature imputation, scaling, or model fitting.

As a statistical control, permutation testing was performed at each cumulative feature step by shuffling the training labels while preserving the held-out subject's test labels. The observed mean balanced accuracy across held-out subjects was compared with the permutation-based null distribution to assess whether cross-subject decoding performance exceeded chance-level performance.

### Permutation and statistical analyses

To assess whether decoding performance exceeded CL, we conducted a permutation test based on label shuffling. For each subject, relative pitch class labels associated with imagery note epochs were randomly permuted while neural features remained unchanged. Decoding models were then trained and evaluated on the permuted data using the same training, validation, and test set procedures applied to the original data.

This permutation procedure was repeated 200 times for each subject to generate a null distribution of decoding accuracies expected under the assumption that neural features were unrelated to the true pitch labels. The decoding accuracy obtained with the true labels was compared against this null distribution, and an empirical *p* value was computed as the proportion of permuted accuracies that exceeded the observed decoding accuracy. This nonparametric approach allowed us to evaluate decoding performance while preserving the temporal and spectral structure of the neural data, thereby providing a robust estimate of CL performance.

### Code accessibility

The code described in the paper is freely available online at https://github.com/JII-Kwon/ECoG_Music_Imagined_Classification. The code is available as Extended Data 1.

10.1523/ENEURO.0289-25.2026.d1 Data 1 **Code accessibility statement.** The computer code included as Extended Data consists of two components. MATLAB scripts were used for data preprocessing, and Python code was used for feature extraction, decoding, and statistical analysis. The code is freely available in an online repository https://github.com/JII-Kwon/ECoG_Music_Imagined_Classification. Download Data 1, ZIP file.

## Results

### Cortical activation during music imagery

To identify cortical regions engaged during music imagery, we first examined task-related changes in HGA relative to baseline fixation. Electrodes were classified as active based on a significant increase in HGA during the imagery phase compared with baseline.

**Figure 3.**
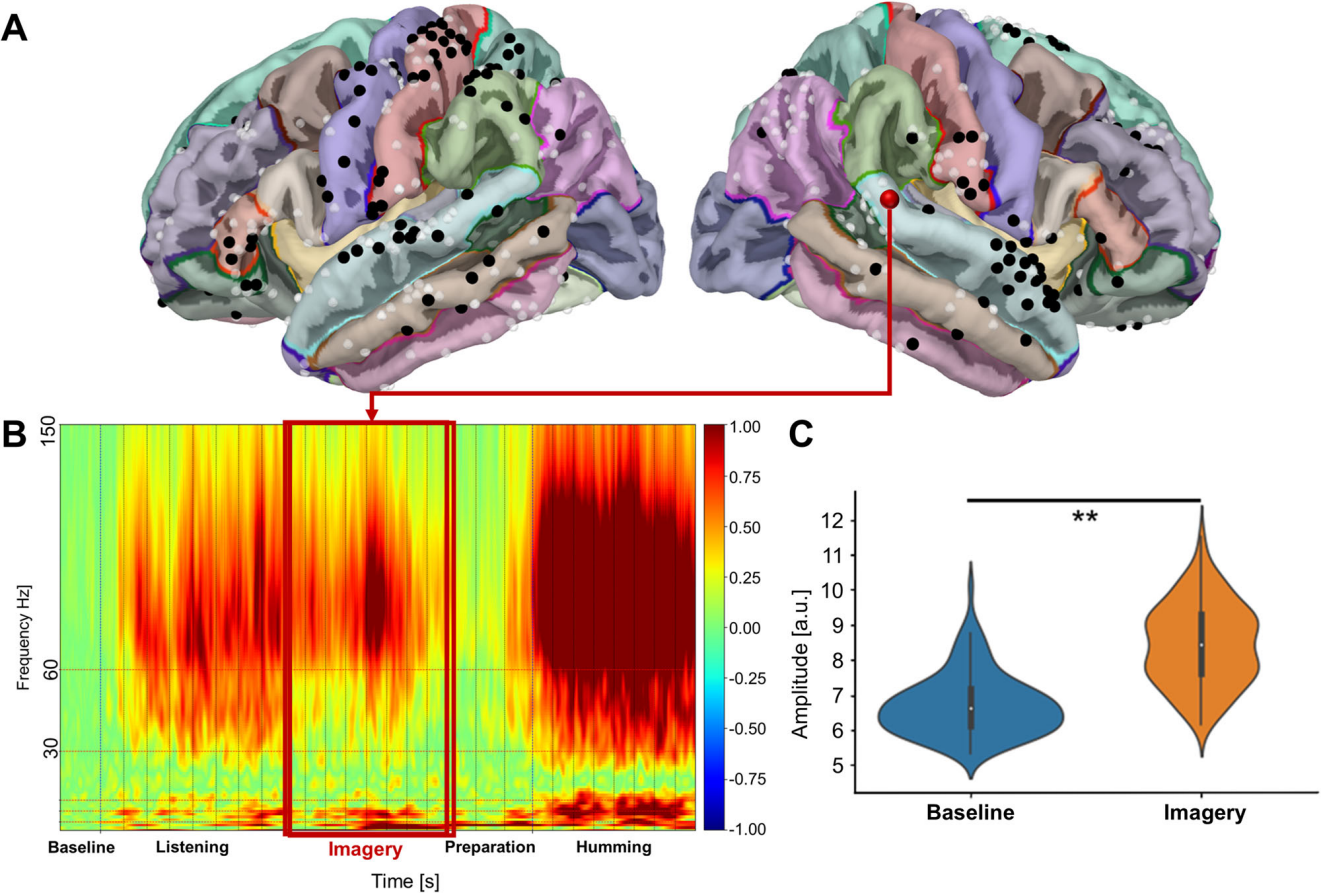
Cortical activation during music imagery. ***A***, Anatomical distribution of imagery-related electrodes projected onto an MNI-standardized cortical surface. Black dots indicate electrodes that exhibited significantly increased HGA during the imagery phase relative to baseline fixation (Wilcoxon signed-rank test, *p* < 0.05). ***B***, Representative time–frequency spectrogram from an electrode located in the left superior temporal gyrus, illustrating task-related changes in spectral power across the full trial sequence (baseline, listening, imagery, preparation, and humming). The imagery period is highlighted in red. ***C***, Group-level distribution of mean HGA values across all active electrodes during baseline and imagery periods. Violin plots show the distribution of HGA values across electrodes, with central lines indicating the median. See Extended Data Figure 3-1 for a sensitivity analysis comparing broadband and multiband PCA-based high-gamma feature extraction methods.

Figure 3*A* shows the anatomical distribution of active electrodes projected onto an MNI-standardized cortical surface. Imagery-related activation was observed across a distributed network, with the highest concentration of active electrodes located in the STG. Additional active electrodes were observed in frontal and parietal regions, including inferior frontal, precentral, and superior parietal cortices. Although the spatial distribution of active electrodes varied across subjects due to differences in electrode implantation, a consistent involvement of temporal lobe regions was observed across subjects.

10.1523/ENEURO.0289-25.2026.f3-1 Figure 3-1 **Sensitivity analysis of high-gamma feature extraction across subjects.** For each subject, channel-wise imagery–baseline effect sizes (Cohen’s d) derived from broadband high-gamma activity (BP-HGA; 61–150 Hz bandpass + Hilbert envelope) are plotted against effect sizes derived from an alternative multi-band PCA-based high-gamma representation (PCA-HGA). Each dot represents one recording channel. Dashed lines indicate the identity line (y = x). Pearson correlation coefficients (r) quantify the agreement of effect-size patterns across channels for each subject. Across subjects, effect sizes show consistently strong agreement and close alignment with the identity line, indicating that the magnitude and spatial pattern of imagery-related HG modulation are preserved across HG extraction methods. Download Figure 3-1, TIF file.

To illustrate the temporal and spectral characteristics of imagery-related activation, Figure 3*B* presents a representative time–frequency spectrogram from an electrode located in the left STG. The spectrogram spans the full task sequence, including baseline fixation, music listening, music imagery, preparation, and humming. During the imagery phase (highlighted in red), broadband HGA was elevated relative to baseline and was temporally aligned with the imagery window, while remaining distinct from the preceding listening phase and the subsequent humming phase.

Group-level quantification of imagery-related activation is shown in Figure 3*C*, which summarizes the distribution of mean HGA values across all active electrodes during baseline and imagery periods. HGA values were significantly higher during music imagery than during baseline fixation (*p* < 0.01, Wilcoxon signed-rank test).

### Intrasubject relative pitch decoding performance

To evaluate whether relative pitch classes could be decoded from music imagery at the single-note level, we assessed intrasubject decoding performance using subject-specific models. Decoding accuracy was evaluated separately for validation data and for an independent test set comprising the final session.

**Figure 4.**
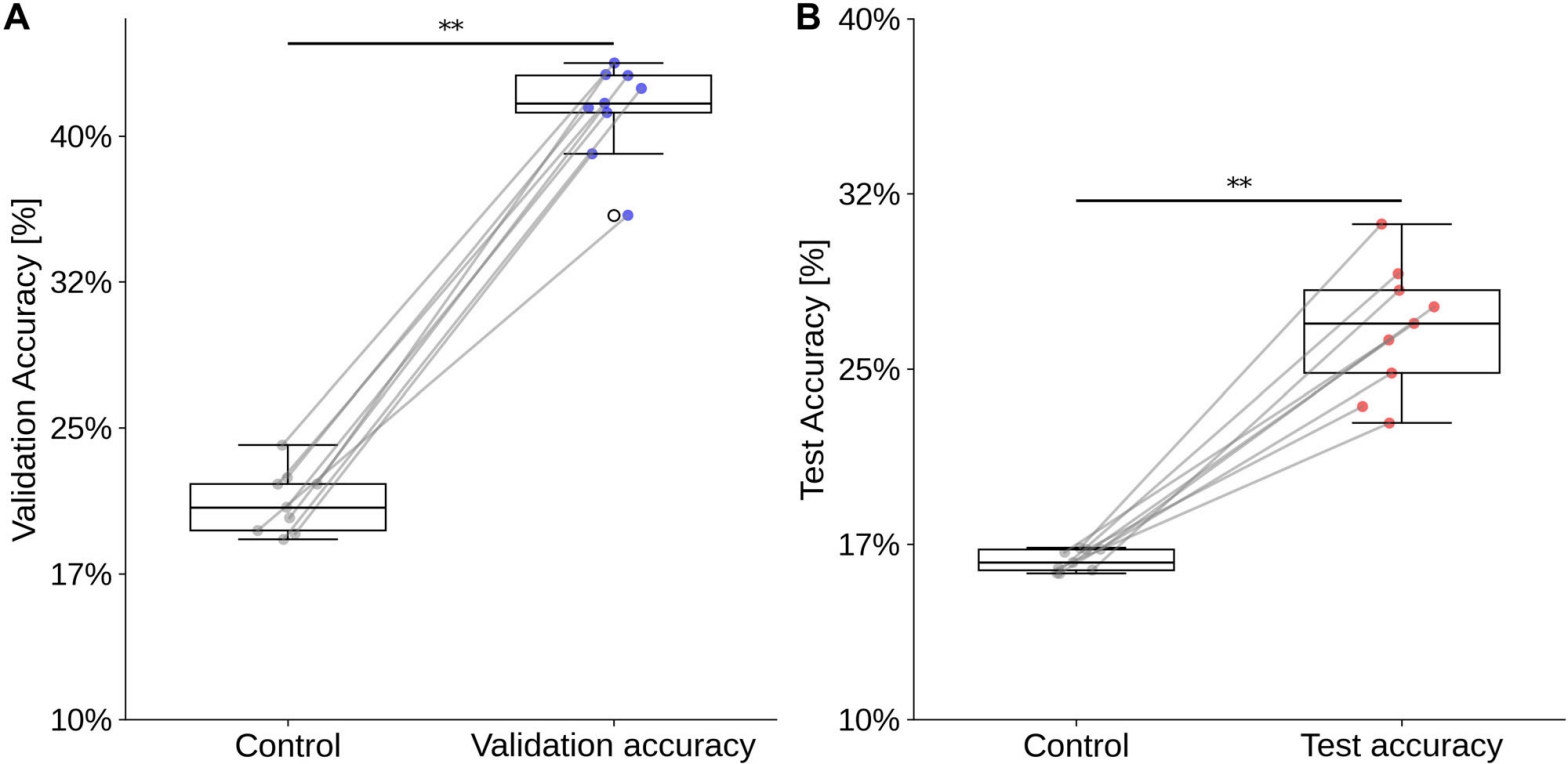
Intrasubject relative pitch decoding performance. ***A***, Validation accuracy of intrasubject decoding models obtained during cross-validation on the training data, compared with a permutation-based control condition. Each dot represents one subject, and gray lines connect paired control and decoding accuracies within subjects. Boxplots summarize the distribution of decoding accuracies across subjects. ***B***, Test accuracy evaluated on an independent held-out test set (final session), compared with the corresponding permutation-based control condition. Each dot represents one subject, with paired control and test accuracies connected by gray lines. See Extended Data Figure 4-1 for absolute pitch decoding performance, Extended Data Figure 4-2 for scale degree decoding within the same absolute pitch height, and Extended Data Figure 4-3 for the control analysis excluding the first two notes of each imagery trial.

Figure 4*A* summarizes validation accuracy across subjects. For each subject, decoding accuracy obtained using the trained model was compared against a control condition in which class labels were permuted. Validation accuracy was significantly higher than the control condition across subjects (*p* < 0.01, Wilcoxon signed-rank test) with a mean accuracy of 41.3%, indicating that the decoding performance exceeded CL.

10.1523/ENEURO.0289-25.2026.f4-1 Figure 4-1 **Absolute pitch decoding performance.** Absolute pitch decoding accuracy compared with chance level (CL = 14.3%). Boxplots represent group-level distributions, with individual subject accuracies shown as paired gray lines. Absolute pitch classification accuracy did not differ significantly from chance across subjects. Classes with fewer than 70 trials were excluded to ensure sufficient sample size per class (C4: 261.63 Hz, D4: 293.66 Hz, E4: 329.36 Hz, F4: 349.23 Hz, F#4: 369.99 Hz, G4: 392.00 Hz, A4: 440.00 Hz). Download Figure 4-1, TIF file.

10.1523/ENEURO.0289-25.2026.f4-2 Figure 4-2 **Decoding performance for notes that shared the same absolute pitch height but differed in scale degree across tonal contexts.** Separate analyses were performed for 2-class and 3-class decoding groups while holding absolute pitch constant within each group. Boxplots represent group-level distributions, with individual subject accuracies shown as gray circles. Violin plots indicate permutation distributions. Decoding performance significantly exceeded the permutation baseline in both the 2-class and 3-class conditions (both p < 0.001), indicating that neural activity contained information sufficient to distinguish scale-degree identities independent of absolute pitch height. Absolute pitch groups included C4 (261.63 Hz), D4 (293.66 Hz), E4 (329.63 Hz), F4 (349.23 Hz), G4 (392.00 Hz), and A4 (440.00 Hz). Download Figure 4-2, TIF file.

10.1523/ENEURO.0289-25.2026.f4-3 Figure 4-3 **Control analysis excluding the first two notes of each imagery trial.** Relative pitch decoding accuracy using the full dataset (“Whole data”) and after excluding the first and second notes from each imagery trial (“DropFirst2”). Boxplots represent group-level distributions, with individual subject accuracies shown as paired gray lines. Decoding accuracy did not differ significantly between conditions (Wilcoxon signed-rank test, n.s.), indicating that the observed decoding performance was not attributable to carryover activity from the preceding listening period. Download Figure 4-3, TIF file.

Generalization of decoding performance was further assessed using the independent test set (final session), as shown in Figure 4*B*. Test accuracy was significantly higher than the corresponding control condition based on label permutation (*p* < 0.01, Wilcoxon signed-rank test). While test accuracy was lower than validation accuracy (mean, 26.7%; maximum, 31.2%), performance remained reliably above chance across subjects.

Together, these results show that relative pitch information during music imagery can be decoded at the single-note level using intrasubject models. At the same time, the modest absolute accuracy highlights the challenges inherent in decoding fine-grained imagined musical content and motivates subsequent analyses at the sequence level.

As a control analysis, we additionally examined whether absolute pitch could be decoded from the same neural features used for relative pitch decoding. Across subjects, absolute pitch classification accuracy did not differ from CL (14.3%; Wilcoxon signed-rank test, n.s.; Extended Data Fig. 4-1). In contrast to relative pitch decoding, which significantly exceeded CL, absolute pitch decoding performance remained indistinguishable from CL in all subjects.

As an additional control analysis, we examined whether notes sharing the same absolute pitch height but differing in scale degree could be distinguished from the same neural features used for relative pitch decoding. Decoding performance remained significantly above chance for both the two-class and three-class conditions (both *p* < 0.001; Extended Data Fig. 4-2). These results indicate that the observed decoding performance cannot be explained solely by absolute pitch height and instead reflects information related to scale degree (relative pitch).

We also tested whether relative pitch decoding was affected by potential carryover activity from the preceding listening period. After excluding the first two notes from each imagery trial, decoding accuracy remained comparable to the original analysis (25.70 ± 4.26% vs 26.71 ± 2.59%; Wilcoxon signed-rank test, n.s.; Extended Data Fig. 4-3).

### Reconstruction of imagined melodies

Here, melody reconstruction refers to a sequence-level representation obtained by temporally ordering independently decoded relative pitch classes for successive imagined notes, rather than to the recovery of acoustic signals or the training of a separate reconstruction model. While single-note decoding accuracy was modest, we next examined whether sequential decoding of relative pitch classes could recover aspects of melodic structure in imagined songs. To this end, imagined melodies were reconstructed by temporally concatenating note-level decoding outputs obtained from intrasubject models.

**Figure 5.**
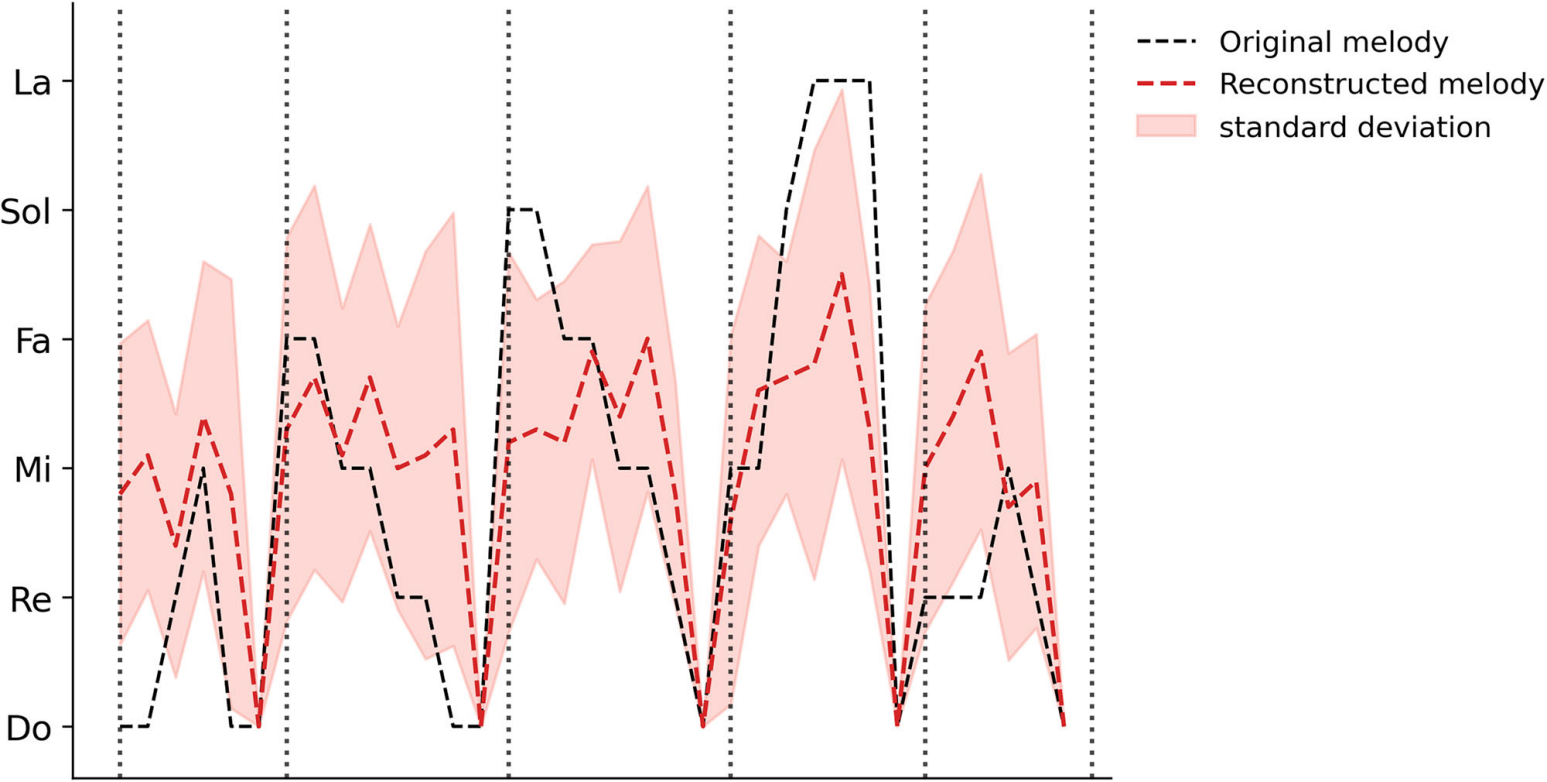
Reconstruction of imagined melodies. Reconstruction of relative pitch sequences for five familiar melodies in a representative subject. The black dashed line indicates the ground-truth relative pitch sequence derived from the musical score, and the red dashed line shows the reconstructed sequence obtained by temporally concatenating note-level decoding outputs. Shaded regions represent the standard deviation of reconstructed relative pitch values across trials. Vertical dotted lines delineate individual melodies. Although deviations occur at the level of individual notes, reconstructed sequences generally follow the overall melodic contour of the original melodies.

Figure 5 and Movie 1 illustrate an example of melody reconstruction for a representative subject across five familiar melodies. For each melody, the ground-truth relative pitch sequence derived from the musical score is shown alongside the reconstructed sequence obtained from decoding. Despite deviations at the level of individual notes, the reconstructed sequences generally followed the melodic contour of the original melodies, reflecting the direction of pitch changes over time.

**Movie 1.** A demonstration of reconstructed imagined melody from brain activity. Demonstration of reconstructed imagined melodies derived from brain signals. The decoding model predicts relative pitch representations from neural activity and enables the reconstruction of the imagined melody. [View online]

Across melodies, reconstruction variability was more pronounced at higher scale degrees, whereas transitions involving the tonic (Do) appeared to be more consistently recovered. Shaded regions indicate the standard deviation of reconstructed pitch values across trials, reflecting trial-to-trial variability in decoding outcomes. Notably, even when individual note predictions were inaccurate, the overall ascending and descending patterns characteristic of each melody were preserved.

To quantify reconstruction performance, we computed the Spearman rank correlation between the reconstructed and ground-truth relative pitch sequences for each melody. The mean reconstruction correlation was 0.67 (*p* < 0.01), indicating a significant correspondence between decoded and original relative pitch sequences.

Together, these results show that relative pitch information decoded at the single-note level can be integrated over time to yield interpretable sequence-level representations of imagined melodies. This reconstruction analysis provides a complementary perspective to single-note decoding accuracy and motivates the examination of higher-order musical structure in subsequent analyses.

### Feature contribution to relative pitch decoding performance

To examine which neural features contributed most strongly to relative pitch decoding performance, we quantified feature-level contributions using the increment value metric derived from sequential feature selection. Increment values reflect the relative increase in decoding accuracy associated with the inclusion of a given feature and therefore provide a measure of feature utility for decoding, rather than direct evidence of neural encoding.

**Figure 6.**
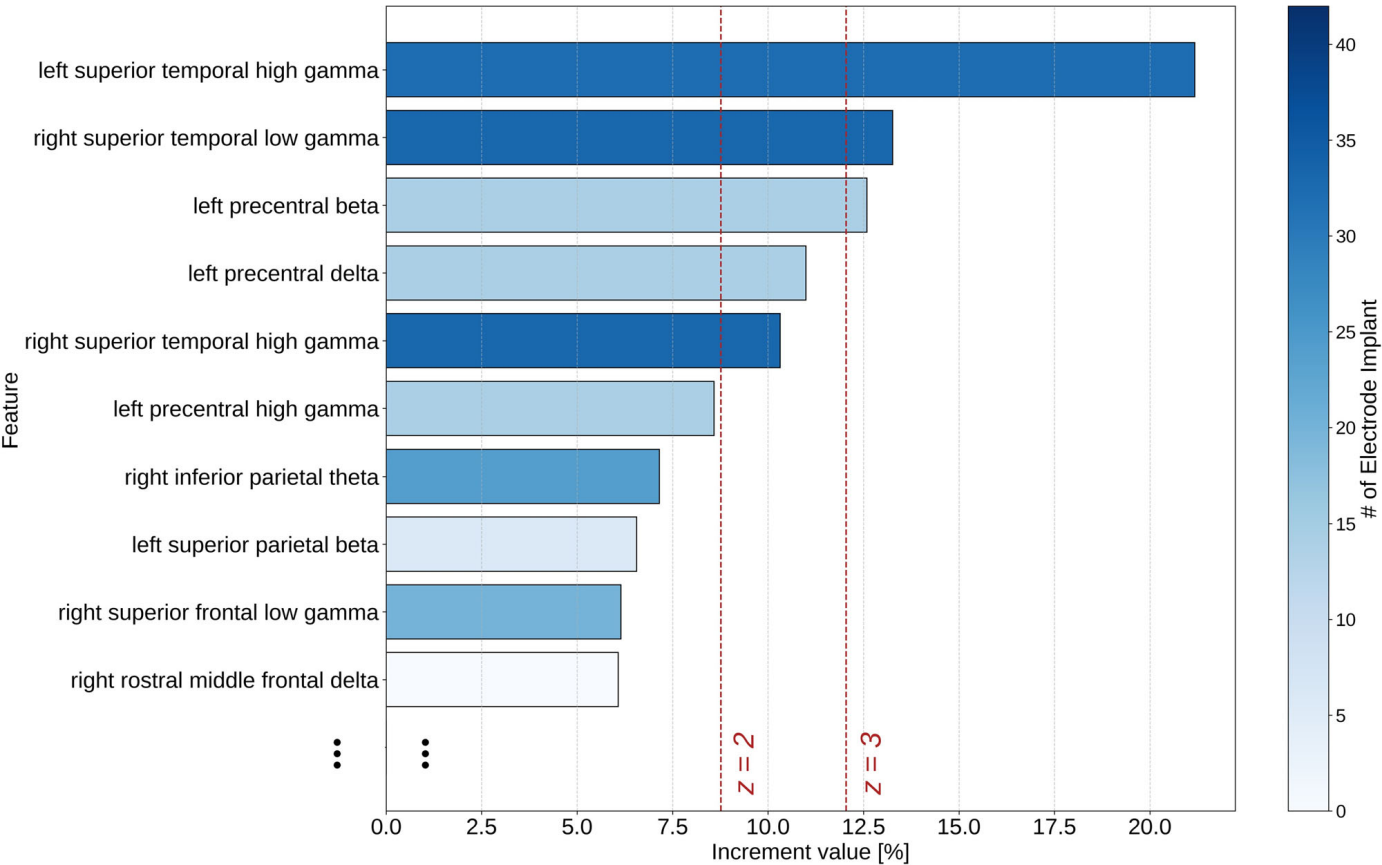
Feature contributions to intrasubject relative pitch decoding. Bar plot showing the top-ranked feature combinations ordered by their mean increment values across subjects. Features are defined by combinations of cortical region and frequency band. Bar length reflects the increment value, indicating the relative increase in decoding accuracy associated with inclusion of each feature during sequential feature selection. Bar color denotes the number of electrode implants contributing to each feature. Dashed vertical lines indicate standardized increment value thresholds (*z* = 2 and *z* = 3). Increment values reflect feature utility for decoding performance and should not be interpreted as direct measures of neural encoding strength. See Extended Data Figure 6-1 for the full distribution of increment values across selected features.

Figure 6 summarizes the top-ranked neural features across subjects, ordered by their mean increment values. The complete set of feature contribution results is provided in Extended Data Figure 6-1. The strongest contributor was HGA activity in the left STG. Additional highly informative features included LGA activity in the right STG and beta-band activity in the left precentral gyrus. Features with moderately high contribution values were also observed in delta-band activity in the left precentral gyrus and HGA activity in the right STG.

10.1523/ENEURO.0289-25.2026.f6-1 Figure 6-1 **Increment value.**This bar graph sorts in descending order based on the magnitude of the increment value for selected features from the best-performing models. The color of each bar represents the total number of implants in the respective area. Abbreviations: D, Delta; T, Theta; A, Alpha; B, Beta; G, Low gamma; HG, High gamma; Download Figure 6-1, TIF file.

These results suggest that relative pitch decoding primarily relied on neural activity in bilateral auditory regions and left motor-related cortex. In particular, the contribution of bilateral STG activity is consistent with the involvement of auditory cortical representations in imagined melody processing, whereas the contribution of left precentral activity may reflect motor-related or subvocal rehearsal components involved in auditory imagery. Although HGA features accounted for the most prominent contribution, informative features were also observed in lower-frequency bands, including beta, delta, and LGA activity. This suggests that relative pitch decoding was supported by spectrotemporal neural features distributed across multiple frequency bands and cortical regions.

The color scale in Figure 6 indicates the number of electrode implants contributing to each feature, highlighting that high-ranking features were supported by data from multiple electrodes rather than driven by isolated contacts. Dashed vertical lines indicate increment value thresholds corresponding to standardized effect sizes (*z* = 2 and *z* = 3), providing a reference for the relative magnitude of feature contributions.

Together, these results identify neural features that consistently enhanced decoding performance across subjects, while emphasizing that increment values quantify contributions to model performance rather than direct neural representations of relative pitch.

### Cross-subject decoding performance

To investigate whether neural features contributing to relative pitch decoding were shared across individuals, we performed a leave-one-subject-out cross-subject decoding analysis using anatomically aligned region-band features. Features were added sequentially according to their ranking in the increment value analysis, and decoding performance was evaluated after each cumulative addition.

**Figure 7.**
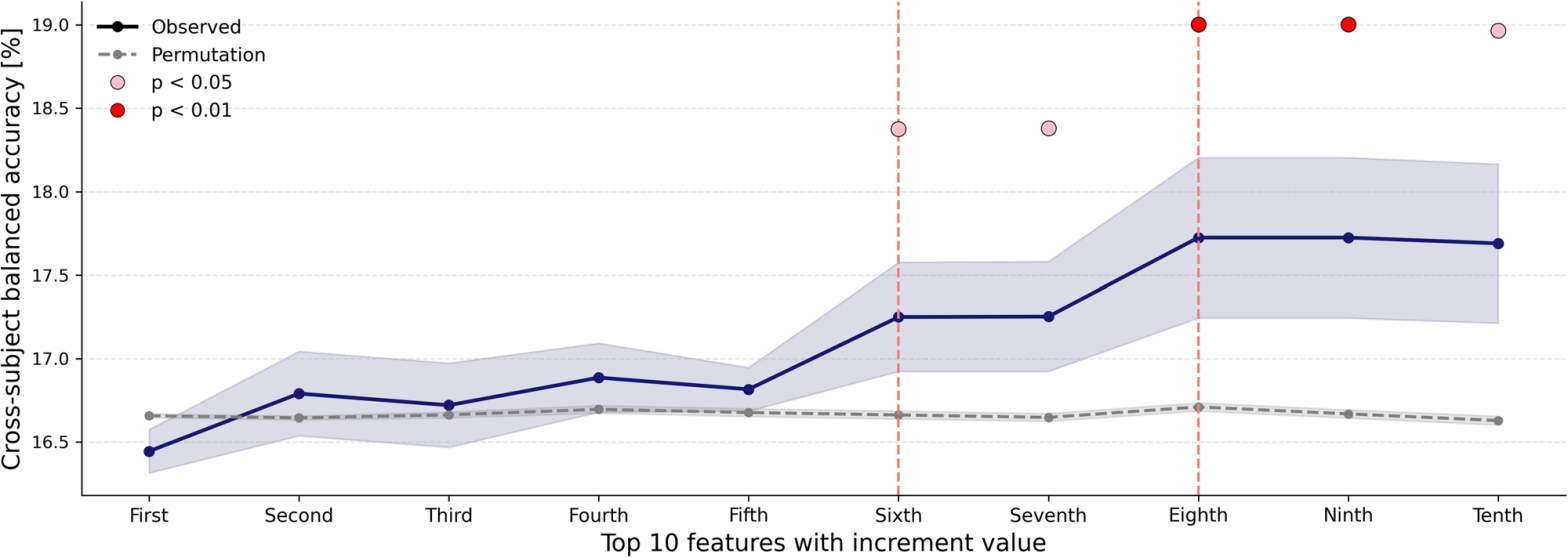
Cross-subject trends in feature contribution to decoding performance. Features were added sequentially according to their ranking in the increment value analysis (Fig. 6), and decoding performance was evaluated using leave-one-subject-out cross-validation. The blue line indicates the observed mean balanced accuracy across held-out subjects, and shaded regions represent ±SE. The gray dashed line indicates the corresponding permutation performance obtained by shuffling training labels, with shaded regions representing ±SE across permutation iterations. Colored circles denote cumulative feature sets that significantly exceeded the permutation-based null distribution (light pink: *p* < 0.05; red: *p* < 0.01). Vertical dashed lines indicate the addition of left precentral high-gamma activity (6th feature) and left superior parietal beta activity (8th feature), which were associated with significant improvements in cross-subject decoding performance.

As shown in Figure 7, decoding performance gradually increased as higher-ranked region-band features were incorporated into the model. The first several features produced only modest improvements relative to permutation performance. However, a more pronounced increase emerged after inclusion of left precentral HGA (6th feature), at which point decoding performance significantly exceeded the permutation-based null distribution (*p* < 0.05). Performance increased further following the addition of left superior parietal beta activity (8th feature), resulting in a stronger level of significance (*p* < 0.01). Comparable performance levels were maintained with the subsequent addition of the remaining features.

These findings suggest that a limited subset of anatomically defined region-band features contributed disproportionately to cross-subject decoding performance. In particular, HGA in the left precentral cortex and beta-band activity in the left superior parietal cortex appeared to provide information that generalized more consistently across individuals than other candidate features. Although overall decoding performance remained modest, the results indicate that specific auditory-motor and parietal features may represent relatively stable neural correlates of relative pitch imagery across subjects.

## Discussion

In the present study, we investigated whether imagined melodies can be decoded from human ECoG recordings using a relative pitch framework. By focusing on familiar melodies and representing melodic content in terms of relative pitch classes rather than absolute pitch, we assessed the feasibility of reconstructing internally generated musical structure using single-note-level decoding as a building block.

We found that relative pitch information during music imagery could be decoded at the single-note level using intrasubject models, with performance reliably exceeding chance. Although single-note decoding accuracy was modest, sequential integration of decoded pitch classes enabled reconstruction of melodic contours that preserved the relative structure of imagined melodies.

Together, these findings provide a proof-of-concept that imagined melodies can be decoded from intracranial neural recordings when melodic content is framed in terms of relative pitch, highlighting the importance of sequence-level approaches for accessing complex internally generated auditory representations.

### Decoding imagined melodies using a relative pitch framework

A central design choice of the present study was to adopt a relative pitch framework for decoding imagined melodies. Relative pitch, defined by the relationships between successive tones, is widely recognized as the primary basis for melody recognition across changes in key and timbre, making it a biologically grounded target for neural decoding (Gómez et al., 2003; Oxenham, 2012).

Absolute pitch could not be decoded from the same neural features, with classification performance remaining at CL across subjects. This dissociation between relative and absolute pitch decoding aligns with behavioral evidence that nonabsolute pitch listeners rely predominantly on interval-based rather than frequency-anchored representations, further supporting the view that relative pitch constitutes the primary neural code underlying imagined melody structure (Leipold et al., 2019; Coy et al., 2021). Consistent with this view, our results show that while single-note decoding accuracy was limited, the sequential structure of decoded pitch classes preserved key aspects of melodic contour.

Because the present study focused on familiar melodies, memory retrieval and rehearsal processes likely contributed to imagery-related neural activity. Accordingly, the findings should not be interpreted as evidence for decoding novel or unconstrained melodic generation. Rather, they demonstrate that relative pitch representations associated with well-learned melodies can be accessed from cortical activity and integrated over time to yield interpretable melodic reconstructions.

### Neural representation of relative pitch in imagery

Feature contribution analyses were conducted to identify neural features that most strongly enhanced relative pitch decoding performance. Importantly, the increment value metric used in this study quantifies the utility of features for decoding accuracy, rather than directly reflecting the strength or locus of neural representations. Accordingly, these results should be interpreted in terms of model performance rather than neural encoding per se.

Across subjects, features derived from auditory cortical regions, particularly the STG, consistently ranked among the strongest contributors to decoding performance. The most prominent contribution was observed for HGA in the left STG, followed by LGA and HGA in the right STG. Additional informative features were identified in beta- and delta-band activity within the left precentral gyrus (Fig. 6). These findings are consistent with the established role of auditory cortex in pitch processing and music imagery (Zatorre et al., 1996; Halpern and Zatorre, 1999; Kraemer et al., 2005).

The observed pattern points to a distributed auditory-motor network supporting relative pitch decoding during music imagery (Meister et al., 2004; Zatorre et al., 2007). Bilateral STG activity may reflect auditory representations of pitch relationships and melodic structure (Zatorre et al., 1996; Patterson et al., 2002), whereas the contribution of left precentral features may be related to motor planning or subvocal rehearsal processes that accompany auditory imagery (Meister et al., 2004; Nishida et al., 2017). Although high-frequency activity accounted for several of the strongest contributors, informative features were also observed in lower-frequency bands, indicating that relative pitch information was represented across multiple spectrotemporal features.

Exploratory cross-subject analyses revealed consistent trends in feature contribution despite substantial interindividual variability in electrode implantation. Rather than indicating subject-independent decoding, these results suggest that certain combinations of spectrotemporal features tend to be informative across individuals, providing a descriptive view of common patterns supporting relative pitch decoding during imagery.

### Methodological considerations and limitations

Several methodological considerations should be taken into account when interpreting the present findings. First, single-note decoding accuracy was modest, reflecting the inherent difficulty of decoding fine-grained content during music imagery, where neural representations are internally generated and temporally variable. Importantly, the present results demonstrate that even under these constraints, sequence-level integration can yield meaningful reconstruction of melodic contour.

Second, the use of familiar melodies likely facilitated stable imagery but limits generalization to novel melodic content. Reconstruction performance was higher for melodies rated as familiar prior to training, suggesting that familiarity supports reliable internal representations. While this effect does not disentangle imagery from memory retrieval, it underscores the importance of well-learned material for decoding and motivates future work using less familiar or novel melodies.

Third, HGA was quantified as broadband power in the 61–150 Hz range, following established conventions in intracranial electrophysiology where broadband HG is commonly interpreted as a robust proxy for population-level cortical activation rather than as a narrowband oscillatory phenomenon. Although averaging across a broad frequency range may obscure finer frequency-specific contributions due to the characteristic 1/f structure of neural power spectra, this choice reflects a deliberate trade-off between robustness, signal-to-noise ratio, and computational efficiency.

To assess the sensitivity of our results to the specific HG extraction method, we performed an additional analysis comparing broadband HG with an alternative multiband principal component analysis-based HG representation that integrates information across multiple sub-bands with different spectral weighting (Oganian and Chang, 2019). Channel-wise imagery–baseline effect sizes derived from the two representations showed strong agreement (*r* = 0.961 ± 0.03), with estimates closely aligned with the identity line (*y* = *x*), indicating that both the magnitude and spatial pattern of task-related HG modulation were highly consistent across methods (Extended Data Fig. 3-1). These findings suggest that the main conclusions of this study are robust to the choice of HG representation and that imagery-related information is preserved when using broadband HG.

Nevertheless, frequency-resolved HG analyses may reveal more fine-grained spectral contributions under different task conditions or analytical objectives. Future work could therefore systematically evaluate the potential benefits of sub-band-normalized or frequency-resolved HG features, particularly in contexts where offline decoding accuracy is prioritized over real-time feasibility.

Fourth, although neural power exhibits nonlinear (approximately logarithmic) scaling properties, several aspects of the present analysis mitigate potential distortions arising from linear treatment of power values. Features were derived from amplitude envelopes obtained via the Hilbert transform rather than from raw power estimates, and baseline *z*-normalization was applied to reduce inter-trial and inter-condition variability. Moreover, the decoding framework relied on a random forest classifier, which is largely insensitive to monotonic transformations of input features, suggesting that explicit log-transformation of power values would be unlikely to qualitatively alter decoding performance or feature ranking. Nevertheless, future studies could explicitly compare linear and logarithmic feature representations to assess their impact on decoding under different modeling assumptions.

Additional limitations include the focus on a single-octave pitch range and the use of temporally averaged neural features within each note window. Extending decoding to multiple octaves and incorporating finer-grained temporal dynamics represent important directions for future research. Finally, in the absence of electromyographic recordings, subtle movement-related activity cannot be entirely ruled out, although subjects were instructed to refrain from overt movement and were continuously monitored throughout the experiment.

### Implications and future directions

The present study provides evidence for the feasibility of decoding imagined melodies from intracranial neural recordings when melodic content is framed in terms of relative pitch. By showing that sequential decoding can preserve melodic contour, this work highlights the importance of representational choices and sequence-level analyses for accessing complex internally generated auditory content.

Future research should extend this framework to novel melodies, broader pitch ranges, and richer temporal representations, as well as explore improved cross-subject alignment strategies. More broadly, these findings contribute to ongoing efforts to move beyond the detection of mental states toward the decoding of structured internal content, informing future investigations of imagined auditory experience.

Attritionary, the cross-subject analysis in the present study highlights the potential of feature-domain representations for aggregating information across individuals while preserving anatomical interpretability. By organizing neural features at the level of shared anatomical regions and frequency bands, rather than individual electrodes, this representation provides a principled foundation for extending decoding frameworks beyond subject-specific models. As such, the present results suggest a potential pathway toward subject-independent or transfer learning-based decoding approaches, in which shared feature domains could be used to initialize, constrain, or adapt models for new individuals with limited data. Future work could explicitly pursue this direction by incorporating domain adaptation or transfer learning techniques on top of the feature aggregation scheme introduced here.

## Data Availability

The data that support the findings of this study are available from the corresponding authors upon request.

## Synthesis

Reviewing Editor: Anne Keitel, University of Dundee Decisions are customarily a result of the Reviewing Editor and the peer reviewers coming together and discussing their recommendations until a consensus is reached. When revisions are invited, a fact-based synthesis statement explaining their decision and outlining what is needed to prepare a revision will be listed below. The following reviewer(s) agreed to reveal their identity: David Quiroga-Martinez, Debadatta Dash.

The manuscript "Reconstruction of Imagined Melody with Relative Pitch Decoding in Electrocorticography" has now been re-reviewed by both original reviewers, and both acknowledged the thorough revision of the manuscript. However, there were some remaining methodological concerns and questions, which are listed below. The decision was therefore "Revise and resubmit". Please respond to each concern in a point-by-point manner.

General assessment for your information (no need to respond)

(1) Music imagery classification is a trending field. The authors used ECoG to successfully decode and reconstruct imagined melody, albeit with low accuracy. The highlight part was their attempt for cross-subject decoding. Thank you for clearly explaining all the queries. The manuscript is significantly advanced.

(2) I want to thank the authors for their thoughtful revision of the manuscript. The text has substantially improved in clarity and readability making it easier to understand what was done. If confirmed, the results would be an interesting addition to the literature on decoding of imagined music from neural activity. However, some critical methodological issues remain that need to be addressed to demonstrate the validity of the findings.

Comments - please respond to these

1. The features were z-scored per tonality using data related to Do on each tonality. This procedure remains as a big potential source of bias because now the Do category has a consistent mean of 0 and an SD of 1, which might introduce systematic differences with other pitch classes. I do not see why this procedure is necessary, because the three tonalities overlap a lot in their pitch range and share common pitch heights, which probably makes the impact of pitch height negligible in decoding. Moreover, it is not clear whether the mean and SD were estimated excluding the test set or not. If not, then there is a big risk of leakage and overfitting. To properly demonstrate decoding of scale degree I suggest the following:

a. Ensuring that the scaling parameters are obtained from the training set.

b. Train decoders to distinguish pitches with the same height but different scale degree.

c. Repeat the current analyses without the normalization procedure.

2. The first few notes of the imagination period might have carryover activity from the listening period, potentially biasing decoding. Would the results change after exclusion of those notes?

3. For the Cross-Subject decoding analysis, it is said that the features were pooled across subjects. Does pooling mean that they were averaged per subject, frequency band and region? Or was only one channel taken per region per subject and frequency band? Please clarify.

## Author Response

Responses to the Reviewers' Comments Manuscript ID: eN-NWR-0289-25 Manuscript Title: Reconstruction of Imagined Melody with Relative Pitch Decoding in Electrocorticography Dear editor and reviewers in eNeuro, We are sincerely grateful to the editor and reviewers for the opportunity to revise and improve our manuscript, "Reconstruction of Imagined Melody with Relative Pitch Decoding in Electrocorticography". We have carefully addressed the reviewers' comments and revised the manuscript accordingly. Below, we provide a point-by-point response to each concern. Changes in the revised manuscript are highlighted in 'Manuscripts_ver_2_0.docx'. [General Evaluation] The manuscript "Reconstruction of Imagined Melody with Relative Pitch Decoding in Electrocorticography" has now been re-reviewed by both original reviewers, and both acknowledged the thorough revision of the manuscript. However, there were some remaining methodological concerns and questions, which are listed below. The decision was therefore "Revise and resubmit". Please respond to each concern in a point-by-point manner.

General assessment for your information (no need to respond) (1) Music imagery classification is a trending field. The authors used ECoG to successfully decode and reconstruct imagined melody, albeit with low accuracy. The highlight part was their attempt for cross-subject decoding. Thank you for clearly explaining all the queries. The manuscript is significantly advanced. (2) I want to thank the authors for their thoughtful revision of the manuscript. The text has substantially improved in clarity and readability making it easier to understand what was done. If confirmed, the results would be an interesting addition to the literature on decoding of imagined music from neural activity. However, some critical methodological issues remain that need to be addressed to demonstrate the validity of the findings.

Author's response:

We sincerely thank the editor and the reviewers for their positive evaluation of our revised manuscript and for acknowledging the improvements made in the previous revision. We also appreciate the constructive comments regarding the remaining methodological concerns. In this revision, we have addressed these points through additional control analyses and methodological clarifications, as detailed in the point-by-point responses below. . [Major Comments] Comment 1:

The features were z-scored per tonality using data related to Do on each tonality. This procedure remains as a big potential source of bias because now the Do category has a consistent mean of 0 and an SD of 1, which might introduce systematic differences with other pitch classes. I do not see why this procedure is necessary, because the three tonalities overlap a lot in their pitch range and share common pitch heights, which probably makes the impact of pitch height negligible in decoding. Moreover, it is not clear whether the mean and SD were estimated excluding the test set or not. If not, then there is a big risk of leakage and overfitting. To properly demonstrate decoding of scale degree I suggest the following: a. Ensuring that the scaling parameters are obtained from the training set. b. Train decoders to distinguish pitches with the same height but different scale degree. c. Repeat the current analyses without the normalization procedure.

Author's response:

We thank the Reviewer for this important comment. We agree that the potential effects of the tonality-specific normalization procedure, pitch-height confounds, and possible data leakage should be carefully addressed. We have therefore clarified the normalization procedure and performed additional control analyses to address each of the Reviewer's concerns. Detailed responses are provided point-by-point below. a. Ensuring that the scaling parameters are obtained from the training set.

Author's response: We thank the Reviewer for this comment. As suggested in Comment 1c, we repeated the analyses without the tonality-specific normalization procedure and adopted these results in the revised manuscript. Because no tonality-specific scaling is now applied, concerns regarding the estimation of scaling parameters and potential information leakage are no longer applicable. Accordingly, we removed the normalization procedure from the Methods section and updated the manuscript throughout to reflect the revised analysis pipeline. b. Train decoders to distinguish pitches with the same height but different scale degree.

Author's response: We thank the reviewer for this helpful suggestion. To determine whether decoding performance reflected scale-degree information rather than absolute pitch height, we performed an additional control analysis using only notes that shared the same absolute pitch but differed in scale degree across tonal contexts. In this analysis, absolute pitch height was held constant within each decoding group.

Decoding performance remained significantly above the permutation baseline for both the 2-class and 3-class conditions (both p < 0.001). These results indicate that notes with identical absolute pitch height, but different scale-degree identities could be reliably distinguished from neural activity. Therefore, the observed decoding performance cannot be explained solely by differences in absolute pitch height and instead supports the presence of scale-degree (relative pitch) information in the neural signals.

The corresponding results have been added to the revised manuscript (Extended Data Figure 4-2).

Related revised manuscript text (or Table/Figure): [Results, line 383 ~ 392, page 18] 3.2. Intra-subject Relative Pitch Decoding Performance ...

As an additional control analysis, we examined whether notes sharing the same absolute pitch height but differing in scale degree could be distinguished from the same neural features used for relative pitch decoding. Decoding performance remained significantly above chance for both the 2-class and 3-class conditions (both p < 0.001; Extended Data Figure 4-2). These results indicate that the observed decoding performance cannot be explained solely by absolute pitch height and instead reflects information related to scale degree (relative pitch). ... [Figure legends, line 802 ~ 805, page 34] Figure 4. Intra-subject relative pitch decoding performance. ... See Extended Data Figure 4-1 for absolute pitch decoding performance, Extended Data Figure 4-2 for scale-degree decoding within the same absolute pitch height, and Extended Data Figure 4-3 for the control analysis excluding the first two notes of each imagery trial. [Extended Data Figure Legends, line 874~883, page 38] Extended Data Figure 4-2. Decoding performance for notes that shared the same absolute pitch height but differed in scale degree across tonal contexts.

Separate analyses were performed for 2-class and 3-class decoding groups while holding absolute pitch constant within each group. Boxplots represent group-level distributions, with individual subject accuracies shown as gray circles. Violin plots indicate permutation distributions. Decoding performance significantly exceeded the permutation baseline in both the 2-class and 3-class conditions (both p < 0.001), indicating that neural activity contained information sufficient to distinguish scale-degree identities independent of absolute pitch height. Absolute pitch groups included C4 (261.63 Hz), D4 (293.66 Hz), E4 (329.63 Hz), F4 (349.23 Hz), G4 (392.00 Hz), and A4 (440.00 Hz). c. Repeat the current analyses without the normalization procedure.

Author's response: We thank the Reviewer for this important suggestion. To directly address this concern, we repeated the current analyses after completely removing the tonality-specific z-score normalization procedure. In the revised manuscript, the main results are now based on this analysis without tonality-specific normalization. Accordingly, we also removed the corresponding methodological description of the tonality-specific normalization procedure from the Methods section.

The decoding and reconstruction performance remained comparable after removing the normalization procedure. The mean validation accuracy slightly increased from 40.4% to 41.3%, and the mean test accuracy increased from 25.5% (max: 30.5%) to 26.7% (max: 32.1%). In addition, the melody reconstruction performance remained highly similar, with the correlation coefficient changing only slightly from r = 0.68 to r = 0.67 (p<0.01).

The feature contribution analysis showed some changes in the top-ranked regions after removing the normalization procedure. However, the most informative features were still observed in auditory and motor-related regions, including HGA activity in the left STG, LGA and HGA activity in the right STG, and beta- and delta-band activity in the left precentral gyrus. These results indicate that the decoding and reconstruction performance did not depend on the original normalization procedure.

Because the feature contribution analysis was recomputed after removal of the normalization procedure, the ranking of the top contributing region-band features changed slightly. In addition, following Reviewer Comment 3, we revised the exploratory cross-subject analysis to use a leave-one-subject-out framework with anatomically aligned region-band features, providing a more conservative evaluation of cross-subject decoding. Accordingly, the cross-subject analysis was repeated using the revised feature ranking and evaluation procedure.

Related revised manuscript text (or Table/Figure): [Results, line 363 ~ 372, page 17] 3.2. Intra-subject Relative Pitch Decoding Performance ...

Figure 4A summarizes validation accuracy across subjects. For each subject, decoding accuracy obtained using the trained model was compared against a control condition in which class labels were permuted. Validation accuracy was significantly higher than the control condition across subjects (p < 0.01, Wilcoxon signed-rank test) with a mean accuracy of 41.3%, indicating that the decoding performance exceeded CL.

Generalization of decoding performance was further assessed using the independent test set (final session), as shown in Figure 4B. Test accuracy was significantly higher than the corresponding control condition based on label permutation (p < 0.01, Wilcoxon signed-rank test). While test accuracy was lower than validation accuracy (mean, 26.7%; maximum, 31.2%), performance remained reliably above chance across subjects. ... [Results, line 413~416, page 19] 3.4. Feature Contribution to Relative Pitch Decoding Performance ... To quantify reconstruction performance, we computed the Spearman rank correlation between the reconstructed and ground-truth relative pitch sequences for each melody. The mean reconstruction correlation was 0.67 (p < 0.01), indicating a significant correspondence between decoded and original relative pitch sequences.... [Results, line 428 ~ 443, page 19~20] 3.4. Feature Contribution to Relative Pitch Decoding Performance ...

Figure 6 summarizes the top-ranked neural features across subjects, ordered by their mean increment values. The complete set of feature contribution results is provided in Extended Data Figure 6-1. The strongest contributor was HGA activity in the left STG. Additional highly informative features included LGA activity in the right STG and beta-band activity in the left precentral gyrus. Features with moderately high contribution values were also observed in delta-band activity in the left precentral gyrus and HGA activity in the right STG.

These results suggest that relative pitch decoding primarily relied on neural activity in bilateral auditory regions and left motor-related cortex. In particular, the contribution of bilateral STG activity is consistent with the involvement of auditory cortical representations in imagined melody processing, whereas the contribution of left precentral activity may reflect motor-related or subvocal rehearsal components involved in auditory imagery. Although HGA features accounted for the most prominent contribution, informative features were also observed in lower-frequency bands, including beta, delta, and LGA activity. This suggests that relative pitch decoding was supported by spectrotemporal neural features distributed across multiple frequency bands and cortical regions. ... [Results, line 453 ~ 472, page 20~21] 3.5. Cross-Subject Decoding Performance To investigate whether neural features contributing to relative pitch decoding were shared across individuals, we performed a leave-one-subject-out cross-subject decoding analysis using anatomically aligned region-band features. Features were added sequentially according to their ranking in the increment value analysis, and decoding performance was evaluated after each cumulative addition.

As shown in Figure 7, decoding performance gradually increased as higher-ranked region-band features were incorporated into the model. The first several features produced only modest improvements relative to permutation performance. However, a more pronounced increase emerged after inclusion of left precentral HGA (6th feature), at which point decoding performance significantly exceeded the permutation-based null distribution (p < 0.05). Performance increased further following the addition of left superior parietal beta activity (8th feature), resulting in a stronger level of significance (p < 0.01). Comparable performance levels were maintained with the subsequent addition of the remaining features.

These findings suggest that a limited subset of anatomically defined region-band features contributed disproportionately to cross-subject decoding performance. In particular, HGA in the left precentral cortex and beta-band activity in the left superior parietal cortex appeared to provide information that generalized more consistently across individuals than other candidate features. Although overall decoding performance remained modest, the results indicate that specific auditory-motor and parietal features may represent relatively stable neural correlates of relative pitch imagery across subjects. [Figure legends, line 828 ~ 837, page 35] Figure 7. Cross-subject trends in feature contribution to decoding performance.

Features were added sequentially according to their ranking in the increment value analysis (Figure 6), and decoding performance was evaluated using leave-one-subject-out cross-validation. The blue line indicates the observed mean balanced accuracy across held-out subjects, and shaded regions represent {plus minus} STE. The gray dashed line indicates the corresponding permutation performance obtained by shuffling training labels, with shaded regions representing {plus minus} STE across permutation iterations. Colored circles denote cumulative feature sets that significantly exceeded the permutation-based null distribution (light pink: p < 0.05; red: p < 0.01). Vertical dashed lines indicate the addition of left precentral high-gamma activity (6th feature) and left superior parietal beta activity (8th feature), which were associated with significant improvements in cross-subject decoding performance. [Discussion, line 515 ~ 530, page 22~23] 4.2. Neural Representation of Relative Pitch in Imagery ...

Across subjects, features derived from auditory cortical regions, particularly the STG, consistently ranked among the strongest contributors to decoding performance. The most prominent contribution was observed for HGA in the left STG, followed by LGA and HGA in the right STG. Additional informative features were identified in beta- and delta-band activity within the left precentral gyrus (Figure 6). These findings are consistent with the established role of auditory cortex in pitch processing and music imagery (Halpern &Zatorre, 1999; Kraemer et al., 2005; Zatorre et al., 1996).

The observed pattern points to a distributed auditory-motor network supporting relative pitch decoding during music imagery (Meister et al., 2004; Zatorre et al., 2007). Bilateral STG activity may reflect auditory representations of pitch relationships and melodic structure (Patterson et al., 2002; Zatorre et al., 1996), whereas the contribution of left precentral features may be related to motor planning or subvocal rehearsal processes that accompany auditory imagery (Meister et al., 2004; Nishida et al., 2017). Although high-frequency activity accounted for several of the strongest contributors, informative features were also observed in lower-frequency bands, indicating that relative pitch information was represented across multiple spectrotemporal features. ... Comment 2:

The first few notes of the imagination period might have carryover activity from the listening period, potentially biasing decoding. Would the results change after exclusion of those notes? Author's response:

We thank the Reviewer for this valuable suggestion. To evaluate the potential influence of residual neural activity from the preceding listening period, we repeated the relative pitch decoding analysis after excluding the first and second notes from every imagery trial. The decoding performance obtained after exclusion (25.70 {plus minus} 4.26%) was comparable to that of the original analysis (26.71 {plus minus} 2.59%), and no significant difference was observed across subjects (Wilcoxon signed-rank test, p > 0.05). These results suggest that the decoding performance was not driven by carryover activity from the listening period. We have included this control analysis in the Supplementary Materials.

Related revised manuscript text (or Table/Figure): [Results, line 389 ~ 392, page 18] ...

We also tested whether relative pitch decoding was affected by potential carryover activity from the preceding listening period. After excluding the first two notes from each imagery trial, decoding accuracy remained comparable to the original analysis (25.70 {plus minus} 4.26% vs. 26.71 {plus minus} 2.59%; Wilcoxon signed-rank test, n.s.; Extended Data Figure 4-3). [Extended Data Figure Legends, lines 885~892, page 37~38] Extended Data Figure 4-3. Control analysis excluding the first two notes of each imagery trial.

Relative pitch decoding accuracy using the full dataset ("Whole data") and after excluding the first and second notes from each imagery trial ("DropFirst2"). Boxplots represent group-level distributions, with individual subject accuracies shown as paired gray lines. Decoding accuracy did not differ significantly between conditions (Wilcoxon signed-rank test, n.s.), indicating that the observed decoding performance was not attributable to carryover activity from the preceding listening period. Comment 3:

For the Cross-Subject decoding analysis, it is said that the features were pooled across subjects. Does pooling mean that they were averaged per subject, frequency band and region? Or was only one channel taken per region per subject and frequency band? Please clarify.

Author's response:

We thank the Reviewer for this question. We agree that the term "pooled" may have been unclear, and we have revised the description to more precisely state that channel-level features were aggregated within each subject into region-band features.

Specifically, the cross-subject decoding analysis used the top-ranked region-band combinations identified from the feature contribution analysis. For each subject, all electrodes belonging to a given anatomical region and frequency band were averaged at the trial level, yielding a single region-band feature for that subject. Thus, we did not select only one representative channel per region-band combination; rather, all available electrodes within that region-band combination contributed to the corresponding feature.

If a subject did not have electrode coverage for a given region-band combination, that feature was treated as missing for that subject. In the leave-one-subject-out decoding procedure, missing values were imputed using the mean estimated from the training subjects only, and standard scaling was also fitted only on the training data. The held-out subject was therefore not used for feature imputation, scaling, or model fitting. We have clarified this procedure in the revised Methods section.

Related revised manuscript text (or Table/Figure): [Materials and Methods, line 291 ~ 315, page 13 ~ 14] 2.12. Cross-Subject Decoding Analysis In addition to intra-subject decoding, we conducted an exploratory cross-subject decoding analysis to examine whether common neural features supporting relative pitch imagery could be identified across individuals.

To address variability in electrode coverage across subjects, channel-level features were aggregated within each subject into region-band features. Anatomical labels were assigned based on the electrode localization procedure described in Section 2.2. For each subject, all electrodes belonging to the same anatomical region and frequency band were averaged at the trial level, yielding a single feature for each region-band combination. Thus, one representative region-band feature was generated by averaging all available electrodes within that region-band, rather than by selecting a single electrode. If a subject did not have electrode coverage for a given region-band feature, that feature was treated as missing.

The region-band features used for this analysis were selected based on the top-ranked features from the increment value analysis. Specifically, the top 10 region-band combinations were entered cumulatively according to their ranking: the model was first trained using the highest-ranked feature, then using the top two features, and so on up to the top 10 features. This cumulative procedure was used to evaluate how decoding performance changed as additional shared region-band features were included.

Decoding performance was evaluated using leave-one-subject-out cross-validation. For each fold, the model was trained on trials from all subjects except one and tested on the held-out subject. Missing region-band features were imputed using the mean estimated from the training subjects only. Standard scaling was also fitted only on the training data and then applied to the held-out subject. Therefore, the held-out subject was not used for feature imputation, scaling, or model fitting.
